# Experiment study on the cross-layer propagation characteristics of hydraulic fracturing cracks in coal roof rocks

**DOI:** 10.1371/journal.pone.0331970

**Published:** 2025-10-03

**Authors:** Maolin Yang, Shuai Lv, Xing Wang, Mao Wang, Sicheng Wang, Yu Meng, Yongjiang Luo

**Affiliations:** 1 Geotechnical Survey Company, CHN Energy Shendong Coal Group, Shenmu, China; 2 School of Mines, China University of Mining and Technology, Xuzhou, China; 3 CHN Energy Shendong Technology Institute, Shenmu, China; 4 State Key Laboratory of Coal Mine Disaster Dynamics and Control, School of Resources and safety Engineering, Chongqing University, Chongqing, China; Henan Polytechnic University, CHINA

## Abstract

Fracture of the overlying roof of a coal seam has become the main method to increase the permeability of coal seam in coalbed methane exploitation. Because the fracture must grow into the coal and through any stone layers, the mechanical property difference between the overlying roof rocks and coal inevitably leads to significant changes in the crack propagation behaviors. In this study, an embedded strain sensor with low cost and simple operation is used to measure the strain in laboratory samples representing the coal seam and roof near the interface for fractures initiated in the roof to obtain the width change of the hydraulic fracture at the interface. At the same time, the propagation behavior of hydraulic fractures at the interface is expressed by combining fracture images and pumping pressure. The results show that a larger horizontal stress difference, larger flow rate, lower coal seam modulus, and smaller intermediate layer thickness are beneficial for the propagation of hydraulic fractures from the roof into the coal seam and the activation of the roof-coal interface. On-site hydraulic fracturing design should fully consider the in-situ stress and coal seam modulus, and design reasonable fracturing drilling location and injection flow. The width of hydraulic fracture in coal seam is larger than that in roof, and increases with the modulus of coal and roof approach, which is beneficial to the migration of proppant in roof-coal seam hydraulic fracture. Therefore, the migration of fracturing proppant in an actual coal seam roof is limited by the width of hydraulic fractures in the roof.

## 1. Introduction

Coalbed methane (CBM) is the main associated gas of coal resources, which is known as unconventional natural gas [1–3] and dangerous gas in coal mines [4–6]. Because of the need for resource use and improved mine safety, it is critical to effectively discharge CBM from the coal seam. However, it is challenging to extract CBM from coal seams because of the extremely low permeability of coal seams [7–9]and the strong adsorption properties of coal [10–12]. Consequently, reservoir reconstruction operation of gas-bearing coal seams has become a key technology for solving the gas drainage problem.

At present, hydraulic fracturing technology is the a successful technology to increase the permeability of rock, which was introduced into coal mine gas drainage in the 1960s and 1970s [13], and it has become a common and effective means to increase permeability of coal measure strata for efficient gas drainage [14,15]. Hydraulic fracturing in a coal seam involves injecting pressurized fracturing fluid with proppants into the coal seam through ground or underground equipment to fracture rocks for better permeability. CBM extraction by fracturing in coal measure strata usually includes two types: (1) fracturing in a coal seam directly and (2) crack propagation into the coal seam by fracturing in the overlying roof of the coal seam [16]. It is easy to construct hydraulic fracturing holes in coal seams with intact and high-strength coal. However, drilling in low-strength coal seams is challenging, and drilling and fracturing the overlying roof layers in soft coal seams is advantageous for maximizing fracturing effectiveness [14,16–18]. Fracture propagation behavior and its influence range are prerequisites of engineering design. And there are two aspects of factors affecting hydraulic fracturing effectiveness: (1) geological factors such as the in-situ stress, mechanical properties of the rock and natural fracture system. (2) operational factors such as pumping parameters and fracturing process [19–22]. Fracturing in the overlying roof of a coal seam requires that cracks propagate from the roof to the coal seam. However, the physical properties, in-situ stress distribution and material properties of the coal rock mass, roof rock mass and interface are obviously different, which means that the propagation of cracks from the roof to coal seam is more complicated than that in the coal rock mass [23,24]. Therefore, in order to better understand and improve the hydraulic fracturing design of CBM reservoirs, it is necessary to study the cross-layer propagation behavior of fractures initiated in the roof.

Previous researches have focused on the influence of flow, in-situ stress, rock strength, porosity, permeability and natural fracture interaction on fracture propagation. Results show that the fracturing cracks in the roof of coal seam expand usually shows the “T” or “I” shape [25–27]. Hydraulic cracks tend to propagate along the interface between weak coal seam and roof, and then “T” and “I” cracks appear instead of straight cracks [26–28]. Natural cracks will also affect the propagation of hydraulic cracks in the roof, and hydraulic cracks usually show penetration, turning, and blocking propagation behaviors [19,29]. In addition, the “stress shadow” effect appears in the multi-cluster hydraulic cracks in the roof, thus inhibiting the expansion of nearby cracks [30]. Nevertheless, this type of research has mainly focused on changes in crack shape and crack length. As an important fracture parameter, fracture width is related to the fluid flow state and fluid pressure in the fracture [31]. Although the fracture width is not very important for roof weakening fracturing, it directly affects the conductivity of the fracture, and also affects the size and distribution of the proppant entering the fracture, which is extremely important for gas drainage [32–34]. In addition, the change in the width of cracks at the coal-rock interface has an important impact on fluid flow, proppant migration, and subsequent drainage. At present, crack width is mainly analyzed by numerical simulations [35–37]. In laboratory experiments, information on the crack width of cast rock samples is also obtained by acoustic emission, CT and Fiber Bragg Grating technology [38–40]. However, it is difficult to obtain the change in crack width by acoustic emission, CT is difficult to use in large specimens, and Fiber Bragg Grating technology is costly as a strain transducer.

In this study, embedded strain sensors were used to obtain the variation law of the crack width direction in the process of coal roof fracturing. By analyzing the distribution characteristics of fractures, the deformation of coal and rock and the change in injection pressure in the hydraulic fracturing process, the effects of in-situ stress difference, flow rate, coal seam modulus and interlayer thickness on fracture propagation, especially on fracture width, are discussed.

## 2. Equipment system and sample preparation

### 2.1. Equipment system

**Fig 1:**
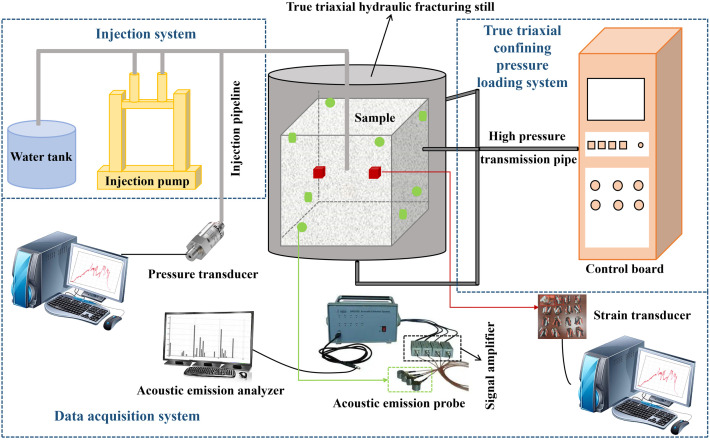
True triaxial rock hydraulic fracturing system.

The equipment system is illustrated in Fig 1. The true triaxial rock hydraulic fracturing system is mainly composed of four systems: the injection system, true triaxial confining pressure loading system, data acquisition system, and true triaxial hydraulic fracturing. The true triaxial confining pressure loading system could exert a maximum pressure of 25 MPa on the fractured samples, and the confining pressure control accuracy was 0.25%. The injection pump is powered by an air compressor and could inject fracturing fluid into the fracturing sample in two ways: constant flow and constant pressure. The maximum constant flow rate is 100 mL/min, and the accuracy is 0.001 ml. The maximum constant pressure is 40 MPa, and the accuracy is 0.1%. The data acquisition system included injection pressure, acoustic emission, and strain collection. The strain acquisition system consists of a strain brick embedded in the fracturing sample and a DHDAS (digital hydraulic displacement and strain) strain acquisition instrument. The material of the strain brick is consistent with a similar material of the stratum, and the size of the strain brick is 10 mm × 10 mm × 10 mm. After the strain brick was cured, an adhesive was used to attach the strain gauge to the strain brick. To prevent the strain gauge from failing in water for similar materials, silicone rubber was used to treat its surface. The DHDAS strain gauge can collect up to 12 groups of data, the sampling frequency can reach up to 1000 Hz, and the strain range is 60000 με with an accuracy 0.1 με.

### 2.2. Sampling

The physical properties of rock layers collected at Xinji No. 2 coal Mine are listed in Table 1. The roof layer is quartz sandstone with a thickness of 0 ~ 35.1 m and the uniaxial compressive strength (UCS) is approximately 10 ~ 20 times that of the uniaxial tensile strength (UTS). The false roof is claystone with a thickness of 0 ~ 0.5 m and its strength is significantly weaker than the roof. The floor is composed of fine sandstone.

**Table 1:** Main layers parameters at the field.

Layers	Average Thickness (m)	UCS (MPa)	UTS (MPa)	Young’s Modulus (GPa)	Poisson’s ratio
Roof	14.9		6.81	15.13	0.11
False Roof	0.3				
Coal	3.28	22.07	2.67	4.41	0.24
Floor	10.8	57.8	4.12	12.87	0.13

**Fig 2:**
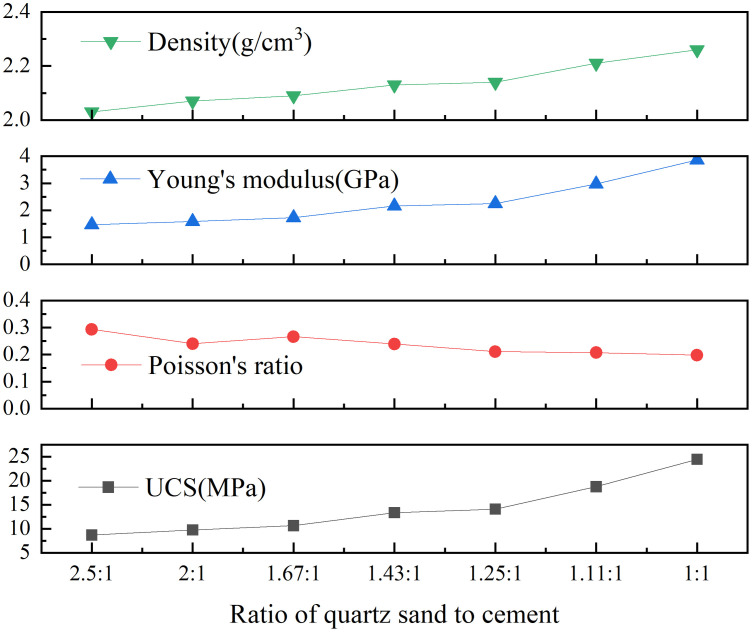
Distribution of physical parameters of similar materials with different proportions.


All samples of the layers were composed of cement and quartz sand in a certain proportion. Uniaxial compression experiments were carried out on repeated standard specimens with quartz sand and cement ratios ranging from 2000: 800 g to 2000: 2000 g previously. The experimental results are shown in Fig 2, the data were analyzed by regression fitting as follows.

$$\begin{array}{c} Y_{\sigma }=4.1511e^{0.0008x},R^{2}=0.9605\\ Y_{\nu }=0.3679e^{-3*10^{-5}x},R^{2}=0.9571\\ Y_{E}=715.94e^{0.0008x},R^{2}=0.9489 \end{array}$$ (1)

where $Y_{\sigma }$: uniaxial compressive strength, MPa; $Y_{v}$: Poisson’s ratio, 1; $Y_{E}$: Young’s Modulus, MPa, x: Quartz sand-cement mixture ratio, 1.

**Fig 3:**
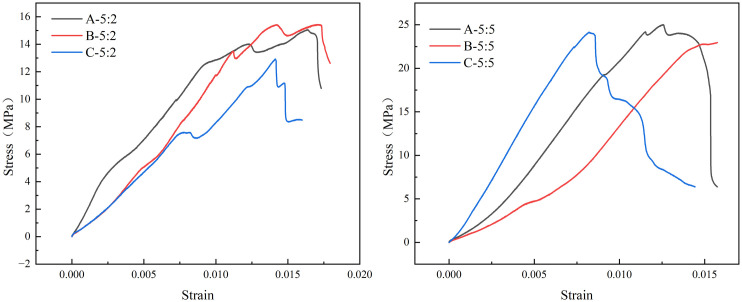
Experimental results of uniaxial compression.

**Fig 4:**
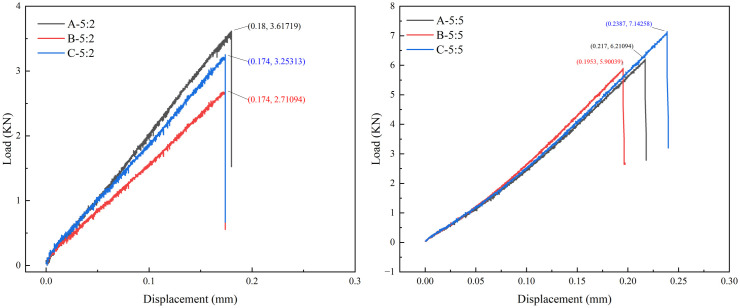
Experiment results of Brazilian test.

It is preliminarily determined that the ratio of sand to cement is 5: 2 and 5: 5 that depends on the strength of laboratory materials and similarity ratio 1: 2 in section 2.3. Rock mechanics tests were performed, and the results are shown in Figs 3 and 4 (Data is from S1 File). When the ratio of quartz sand to cement is 5: 2, the average uniaxial compressive strength, average Young’s Modulus and uniaxial tensile strength are 14.75 MPa, 0.91 GPa and 1.42 MPa respectively. When the ratio of quartz sand to cement is 5: 5, the average uniaxial compressive strength, average Young’s Modulus and uniaxial tensile strength are 24.2 MPa, 2.42 GPa and 3.11 MPa respectively. The test results show that there are some differences between the rock parameters of the specimen and the previous fitting results, which may be caused by differences in the actual sample preparation environment, materials, and technology.

**Fig 5:**
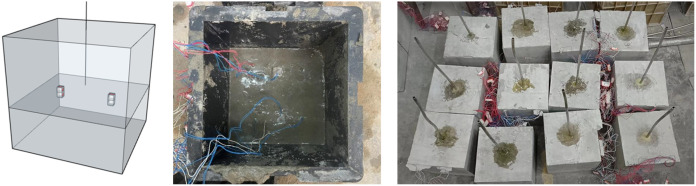
Layout of embedded strain brick and actual drawing of samples.

As Fig 5, the hydraulic fracturing samples were cubes with dimensions of 200 mm × 200 mm × 200 mm. The lower layer of the hydraulic fracturing samples is made of similar material of coal seam with a thickness of 6 cm and a sand-to-cement ratio of 5: 2, and the upper layer is made of similar material of sandstone with a thickness of 14 cm and a sand-to-cement ratio of 5: 5. After the similar materials of the lower coal seam are poured, ensure that the materials are flat. The strain bricks are embedded above the similar materials of the lower coal seam in the right direction, and then the similar materials of the upper sandstone are poured. During the pouring process, ensure that the position of the strain bricks remains unchanged. The samples were cured in a cool and ventilated place for 28 days to obtain the ideal rock strength.

### 2.3. Experiment

Because it is extremely difficult for similar materials to be completely similar to all the physical and mechanical properties of hydraulic fracturing, this study ignores the secondary similarity conditions and only considers similar conditions that have a great influence on hydraulic fracturing. According to the principle of similarity:


$$c_{p}=c_{\sigma H}=c_{\sigma h}=c_{\sigma v}=c_{t}=c_{E}$$ (2)

Where, $c_{p},c_{\sigma H},c_{\sigma h},c_{\sigma v},c_{t},c_{E}$ represent pressure, maximum horizontal stress, minimum horizontal stress, vertical stress, tensile strength and Young’s modulus similarity ratio respectively, and $c_{p},c_{\sigma H},c_{\sigma h},c_{\sigma v},c_{t},c_{E}=2$ in this experiment. Therefore, the parameters of the control experiment are 6 MPa, 3 MPa and 8 MPa for maximum horizontal stress, minimum horizontal stress and vertical stress. In addition, to discuss the influence of in-situ stress, flow rate, coal seam modulus and interlayer thickness variables on the crack width, nine experimental groups were designed, as shown in Table 2. The minimum horizontal stress is allowed to vary in the range of 2 ~ 4 MPa. The influence of the coal seam strength is reflected by Young’s Modulus. Using experimental groups (# 1, # 2 and # 3), (# 2, # 4 and # 5), (# 2, # 6 and # 7) and (# 2, # 8 and # 9), the effects of strain, coal seam strength, flow rate and interlayer thickness on hydraulic fracturing cracks are analyzed respectively. At the same time, the laboratory fracturing fluid is prepared with 0.4% guar gum, and its viscosity is controlled at 35 mPa·s.

**Table 2:** Design of the experimental group.

Designation	σv (MPa)	σH (MPa)	σh (MPa)	E for coal (GPa)	Q (mL/min)	Interlayer thickness (cm)
#1	8	6	2	0.9	60	0
#2	8	6	3	0.9	60	0
#3	8	6	4	0.9	60	0
#4	8	6	3	0.5	60	0
#5	8	6	3	1.6	60	0
#6	8	6	3	0.9	45	0
#7	8	6	3	0.9	75	0
#8	8	6	3	0.9	60	2
#9	8	6	3	0.9	60	4

In the experiment, the specimen is put into the fracturing cylinder first, and then the confining pressure oil pipe, strain line and fracturing pipe are connected. After that, cover the top plate and load the confining pressure according to the preset confining pressure through the true triaxial confining pressure loading system. When the confining pressure is stable, start the strain monitoring system, and at the same time start the injection system to pump fracturing fluid into the specimen for fracturing experiment. During the experiment, the pump pressure is monitored by the system in real time.

## 3. Result and analysis

Limited by the size between the interface and strain brick, the strain in the coal and rock near the interface was collected. Crack propagation is perpendicular to the direction of the minimum principal stress; therefore, the direction of the crack width can be clearly defined as the direction of the minimum principal stress. Meanwhile, the distribution of cracks after fracturing were collected to analyze the change in crack width. The effects of the horizontal stress difference, injection rate, coal seam modulus, and interlayer thickness on the crack width is analyzed respectively.

### 3.1. Influence of in-situ stress on hydraulic fracture width near the interface

**Fig 6:**
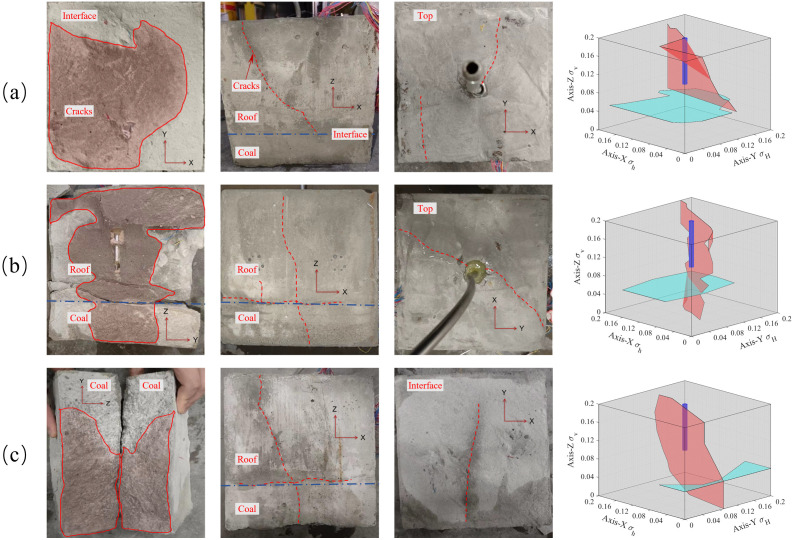
Distribution of hydraulic fractures with different minimum horizontal stresses. (a) Minimum horizontal stress of 4 MPa; (b) Minimum horizontal stress of 3 MPa; (c) Minimum horizontal stress of 2 MPa.

The magnitude of in-situ stress is the main controlling factor for whether hydraulic fractures can cross the coal-rock interface and also affects the width of the hydraulic fractures at the coal-rock interface. In the experimental group, the minimum horizontal in-situ stress in the X direction, the maximum horizontal in-situ stress in the Y direction, and the fracturing fluid flow rates of samples #1, #2, and #3 are the same. The minimum horizontal in-situ stresses were set as 2 MPa, 3MPa, and 4 MPa. The crack propagation shapes after fracturing are shown in Fig 6 (Data is from S2 File).

As shown in Fig 6, when the minimum horizontal stress was 4 MPa, the hydraulic fracture ran through the entire quartz sand layer, and the bedding plane was mostly activated and extended to the boundary. However, the hydraulic fractures did not extend to the coal layer. When the minimum horizontal stress was 3 MPa, the hydraulic fractures ran through the entire quartz sand and coal layers, and some interface planes were activated by the hydraulic fractures. When the minimum horizontal stress was 2 MPa, after the hydraulic fracture initiated from the quartz sand layer, it propagated through the coal seam along the direction of the maximum principal stress, whereas the hydraulic fracture at the interface only expanded near the main cracks. In addition, the included angle between the extension direction of the hydraulic cracks and the direction of the maximum stress (Z axis) in #3 is approximately 30°, while the included angle between the extension direction of the hydraulic cracks and the direction of the maximum principal stress in #1 is approximately 10°. It can be seen that with a decrease in the minimum principal stress, the stress difference increases, resulting in the crack propagation direction tending to extend further along the direction of the maximum principal stress for the coal layer and interface. However, with a further increase in the stress difference, the depth and speed of the cracks entering the coal seam are accelerated, which leads to a decrease in the flow at the interface and cracks propagating only at the edge of the interface.

**Fig 7:**
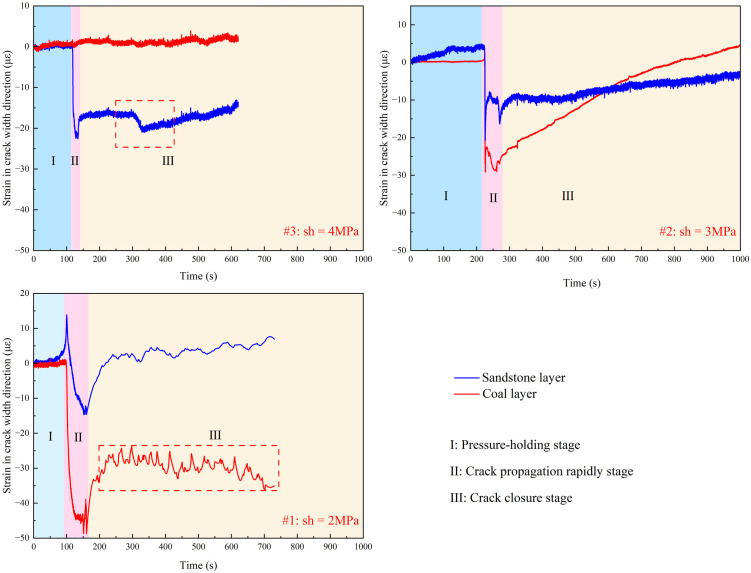
Strain in the direction of the width of the hydraulic crack at the interface of different minimum horizontal principal stresses.

The strain in the crack width direction (X-axis) shows significant differences between experiments #1 and #3 experiments, as shown in Fig 7 (Data is from S2 File). The tensile strain is defined as negative, and compressive strain is defined as positive, with the following consistent definitions. Here, fracturing is divided into three stages: (I) the pre-fracturing stage, which includes the start of data monitoring and the pressure-holding process of the equipment and pipelines. Because of the objective existence of operational errors in the actual operation, this stage is of little significance for discussing the law of fracture propagation; (II) the crack initiation stage, which is the stage of drilling and rock formation pressure suppression and crack propagation; and (III) the crack closure stage, which mainly occurs when the crack runs through the specimen to release pressure and crack closure.

In the crack initiation stage, it is shown that with a decrease in the minimum horizontal stress, the tensile strain of the specimen increases because of the increase in stress difference induced cracks expand to increase stress, which results in the crack propagation stage being lengthened synchronously. However, when the fracture enters the coal layer, the strain of the sandstone layer stratum decreases, and the cracks in the coal seam share the hydraulic pressure and produce wider hydraulic cracks, which proves the result of the above fracture propagation.

**Fig 8:**
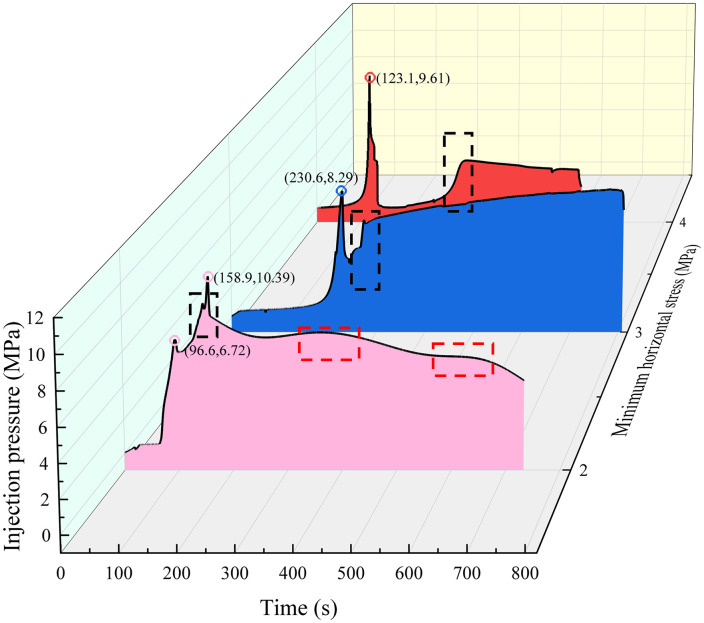
Injection pressures at different minimum principal stresses.

In the crack closure stage, the hole pressure is reduced, and the crack closes when it penetrates through the specimen. In the fracture closure stage of sample #3 with the $\sigma_{h}$ of 4 MPa, although the loss of pore fluid leads to the first closure of the fracture, the fracture only propagates in the sandstone layer, and the area where the fracture breaks through the specimen is not enough to completely relieve the pore pressure, and the tensile strain increases again after the first closure which can be proved by crack turning point of X-Y plane in Fig 6 and the second increase of injection pressure shown as black dotted box in Fig 8 (Data is from S2 File). In sample #2 with the $\sigma_{h}$ of 3 MPa, although the hydraulic fractures in the coal layer share part of the pore pressure, the cracks in the coal layer close more quickly with the weak mechanics properties of the coal layer, which leads to the ineffective weakening of the pore pressure in the sandstone layer and interface gives rise to the slow closure of the cracks proved by the pressure increase slowly after black dotted box in Fig 8. At the same time, the activation of the interface prevents new cracks from appearing in the sandstone; instead, the area activated by the interface expands slowly. In sample #1 with the $\sigma_{h}$ of 2 MPa, the stress difference makes the cracks further enter the coal seam, sharing more hydraulic pressure, and at the same time making it more difficult to close the cracks in the coal seam, and with the continuous generation or reopening of small new cracks results to the pressure drop is wavy in red dotted box of Fig 8. At the same time, the interface fails to extend further because the hydraulic pressure is shared so much that the cracks in the interface only expand near the main cracks.

### 3.2. Influence of injection rate on hydraulic fracture width near the interface

Hydraulic fracturing injects a large amount of fracturing fluid into a coal measure stratum in a short time. When the injection speed of the fracturing fluid exceeds the absorption speed of the formation, the fracturing fluid forms a high pressure at the bottom of the well, resulting in the destruction of the stratum. Therefore, the hydraulic fracturing flow rate also affects the initiation of hydraulic fractures. Fig 10 shows the fractured samples with fracturing fluid injection rates of 45 mL/min, 60 mL/min and 75 mL/min under the same three-dimensional in-situ stress, the same number of strata, and modulus of the specimens.

**Fig 9:**
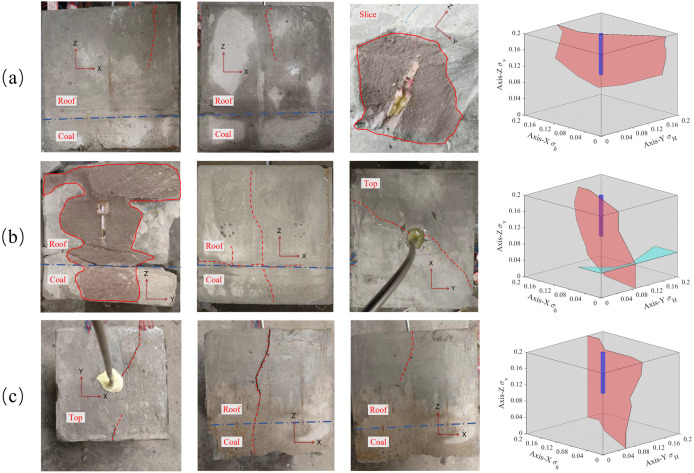
Distribution of hydraulic fractures with different injection rate. (a) injection rate is 45 mL/min; (b) injection rate is 60 mL/min; (c) injection rate is 75 mL/min.

For injection rate of 45 mL/min, hydraulic fractures only extend in the sandstone layer in the vertical direction, and do not extend into the coal-rock interface and coal seam. In the X direction, the hydraulic fracture only extends around the borehole and does not extend to the lateral boundary in the slice shown in Fig 9(a). Obviously, an injection rate of 45 mL/min was not sufficient to cause the hydraulic fracture to spread to the coal layer. Hydraulic cracks extended vertically from the sandstone layer, and extended to the surface of the specimen in the Z and Y directions of the hydraulic cracks at an injection rate of 70 mL/min. An increase in the fracturing fluid flow rate has a particularly significant effect on the extension of hydraulic fractures in the length direction.

**Fig 10:**
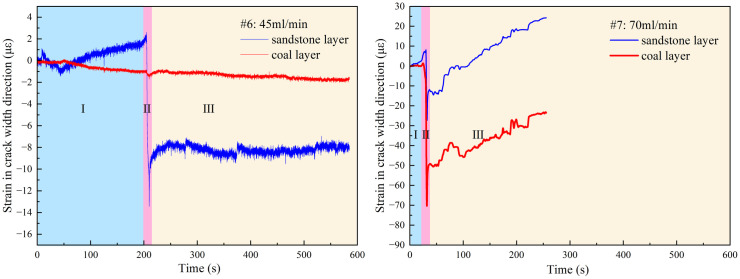
Strain in direction of hydraulic crack width at different injection rates.

In the crack initiation stage, for an injection rate of 45 mL/min, the fracturing fluid pressure in the borehole reached the fracturing pressure, and the hydraulic power extended vertically to the sandstone layer near the interface in the middle of the sandstone layer, as shown in Figs 9 and 10 (Data is from S3 File). At this time, the strain brick in the sandstone layer was compressed in the X direction, and the strain decreased from +2 με to −13 με. There is no obvious rock fracture in the coal seam during the crack propagation stage; therefore, it can be seen that the hydraulic crack does not extend into the coal seam, but it is deformed under the three-dimensional stress load. At an injection rate of 60 mL/min, the cracks crossed the interface and entered the coal seam, as described in Section 3.1.

**Fig 11:**
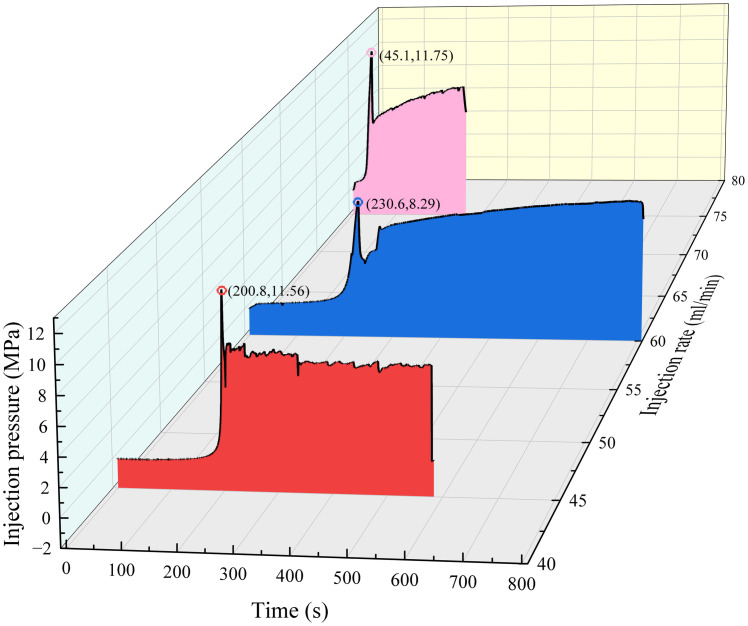
Injection pressures at different injection rates.

At the crack-closure stage, the hydraulic fracture broke through the upper surface of the specimen at 45 mL/min, the fracturing fluid pressure decreased, and the hydraulic fracture began to close. The strain in the width direction of the hydraulic fracture in sandstone increased from −13 με to −7.9 με. However, in contrast to the closing and generation of cracks affected by in-situ stress, the leakage of fracture fluid does not completely reduce the fracturing pressure, but is accompanied by the continuous generation and reopening of small fractures, as shown in Fig 11 (Data is from S3 File). At 75 mL/min, the strong injection rate caused the fracture to completely penetrate the specimen while maintaining a slow increase in injection pressure. At the same time, it can be seen in Fig 10 that the strain of the coal and sandstone layer is still fluctuating and new fractures still occur, which also shows that the injection rate of 75 mL/min is sufficient to meet the hydraulic fracturing of 200 mm specimens in this study. Under certain in-situ stress and injection flow conditions, some small hydraulic fractures may not be observed from the injection pressure.

### 3.3. Influence of coal layer modulus on hydraulic fracture width near the interface

To explore the influence of coal layer modulus on hydraulic fracture, three similar materials, quartz sand and cement ratios are 5: 1, 5: 2 and 5: 3 with the Young’s modulus of 0.5 GPa, 0.9 GPa and 1.6 GPa respectively were used to explore the influence of different coal seam modulus on hydraulic fracture propagation.

**Fig 12:**
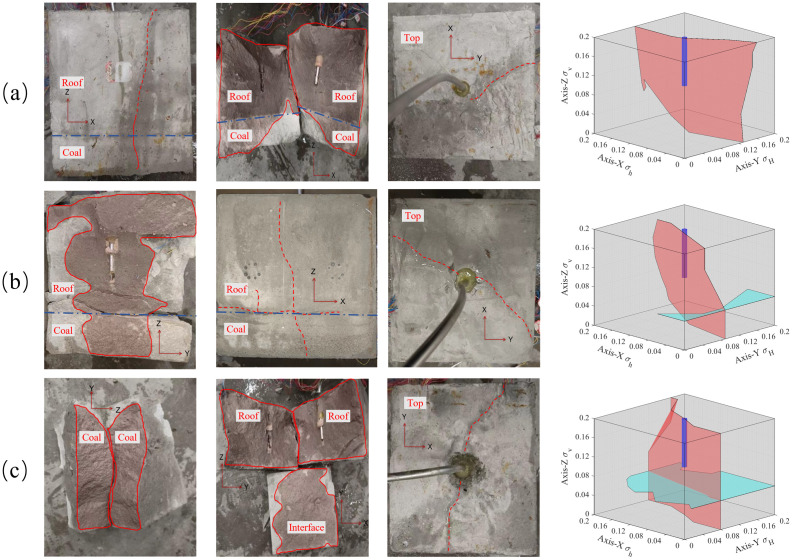
Distribution of hydraulic fractures with different coal layer modulus. (a) E = 0.5 GPa; (b) E = 0.9 GPa; (c) E = 1.6 GPa.

As shown in Fig 12(a) (Data is from S4 File) with a Young’s modulus of 0.5 GPa for the coal layer, the hydraulic fracturing starts from the sandstone layer and extends to the coal layer. The hydraulic fracture easily extends rapidly in the coal layer with low modulus, and the fracturing pressure is maintained at a low level, which is not sufficient to activate the sandstone-coal layer interface. The roof-coal interface was not destroyed. When the modulus of the coal seam increases for E = 0.9 GPa, it becomes difficult for hydraulic fractures to enter the coal seam, and the sandstone-coal interfaces where cracks are linked are activated. When E = 1.6 GPa, the hydraulic fractures extend to the boundary along the Z and Y directions, running through the entire coal layer shown in Fig 12(c) (left), and the interface is almost completely activated, as shown in Fig 12(c) (Data is from S4 File). With the increase of coal seam modulus, it is more difficult for hydraulic fractures entrance into coal layer through the sandstone-coal interface, and the hydraulic fracture are more likely active the sandstone-coal interface, then form “T” shape cracks. Therefore, from the perspective of fracture propagation in hard coal seams, it is best to drill and fracture directly in a high- modulus coal seam to ensure the effective expansion of hydraulic fractures in the coal seam, rather than fracturing in the roof.

**Fig 13:**
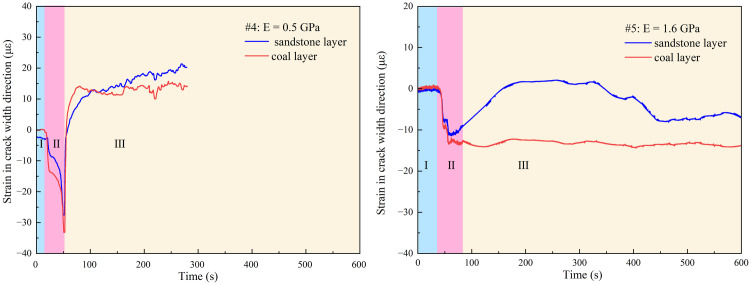
Strain in the direction of the hydraulic crack width at different coal layer modulus.

In the crack initiation stage, comparing Figs 7 and 13, it can be observed that with the increase in coal layer modulus, the difference in the crack width of the coal and sandstone layers decreases. For sample #4 sample, the difference in the coal layer and sandstone layer modulus led to greater deformation of the coal layer than the sandstone layer, resulting in a sharp increase in the width of the hydraulic fracture entering the coal layer. However, compared with specimen #2 specimen which had a modulus of 0.9 GPa, the lower coal seam modulus did not result in a significant increase in the peak tensile deformation of the coal layer. This shows that when the modulus is 0.9 GPa, the deformation and crack width of the coal seam reach the limit under the joint action of the coal seam modulus and in-situ stresses, which in turn causes further deformation and crack opening of the sandstone layer, as shown in Fig 13 (Data is from S4 File). With the close modulus of the coal and sandstone layers, the widths of the hydraulic fractures in the coal and sandstone layers are close to the same, and the deformation of the coal and sandstone layers shows lower tensile deformations and smaller differences, which are determined by the nature of the rock mechanical modulus. In other words, with the decrease in coal seam modulus, the width of the hydraulic fracture in the coal seam increases significantly (in specimens #2 and #5), and the difference between the width of the hydraulic fracture in the coal seam and roof increases, which is beneficial to the migration of the proppant in the fracture. Simultaneously, for extremely weak modulus coal seams, when the crack width of the coal seam reaches the limit, the crack width of the rock stratum increases, and the proppant size allowed to enter the crack increases. Therefore, the appropriate proppant size should be selected according to the modulus of the coal and rock and the in-situ stress situation.

**Fig 14:**
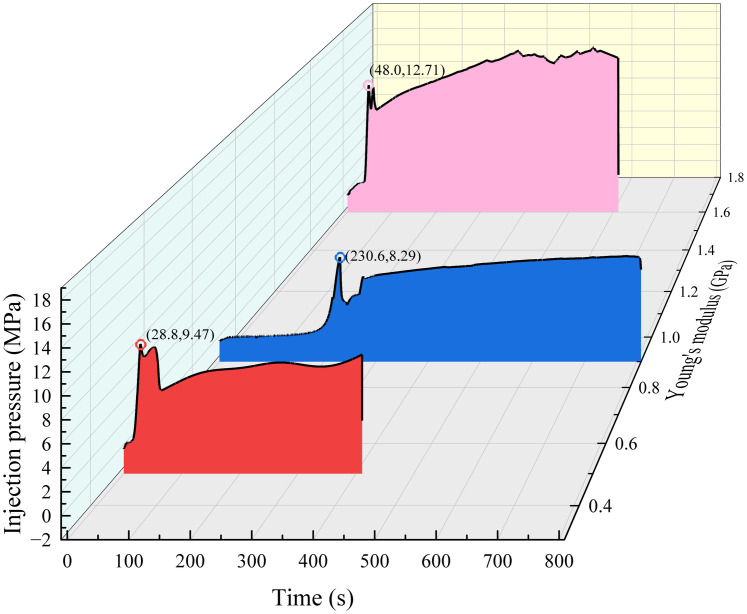
Injection pressure at different coal-layer modulus.

In the crack closure stage, the deformation of the lower-modulus coal layer increased, the crack closure was faster, and even the entire specimen was compressed when the strain was transformed into compression deformation. Meanwhile, the medium-modulus coal seam survey mainly focused on the rapid closure of coal layer cracks, as shown in Fig 7. However, both the cracks in the coal layer and the cracks in the whole layers were closed quickly, and the injection pressure was not completely relieved but slowly increased. Even in sample #4 sample, the small cracks in the coal layer were reopened, which is the strain fluctuation in Fig 13 and the wavy water injection pressure in Fig 14 (Data is from S4 File). When the modulus of the coal layer was close to that of the sandstone layer, the hydraulic fracture in the sandstone layer closed quickly, which led to an increase in the water injection pressure, resulting in the hydraulic fracture opening again. However, the tensile strain in the coal layer was not significantly reduced and the cracks remained open. From the analysis of hydraulic fracture propagation behavior, the proximity of rock modulus makes it easier for hydraulic fractures to be captured by the interface, but the weakness of the interface modulus also makes the fracture easier to close. This leads to two conclusions. First, the coal layer cracks were divided, the fracture fluid was difficult to release, deformation of the coal layer remained stable, and the cracks remained open. Second, the sandstone layer near the fracturing hole becomes the main pressure-relief layer, and the hydraulic fracture in the sandstone layer closes quickly under the combined action of in situ stress and fracture closure, resulting in the injection pressure rising again and the fracture opening again. There is no new crack here, because the crack has no obvious turning point in Fig 12, and the tensile strain rises slowly instead of the sawtooth fluctuation in Fig 13.

### 3.4. Influence of interlayer thickness on hydraulic fracture width near the interface

In field production, the horizon where the borehole is located may not be adjacent to the target coal seam, and there may be thin interlayers with different thicknesses and lithologies, whereas the interlayer may block and deflect the hydraulic fractures. In order to explore the influence of interlayer thickness on hydraulic fracture propagation, interlayers with thicknesses of 0 cm, 2 cm and 4 cm were set on #2, #8 and #9 samples respectively, and the similar materials used for interlayers were sand, cement and water with a ratio of 5: 3: 2.

**Fig 15:**
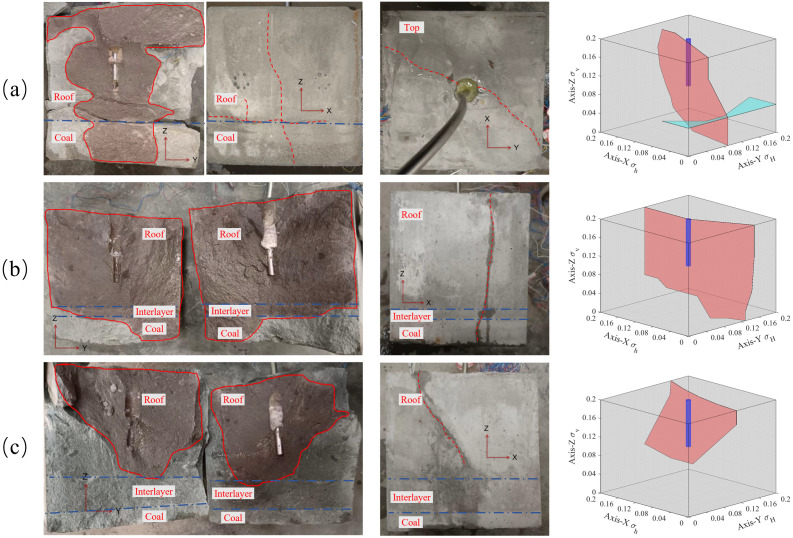
Distribution of hydraulic fractures with different interlayer thicknesses. (a) interlayer thickness of 0 cm; (b) interlayer thickness of 2 cm; (c) interlayer thickness of 4 cm.

With an increase in the interlayer thickness, the possibility of hydraulic cracks spreading from the roof to the coal layer decreased, and hydraulic cracks tended to spread in the roof. As shown in Fig 15(a) (Data is from S5 File), without the interlayer, hydraulic cracks expand into the coal layer by approximately 50% and activate the interface between the coal and roof. When the interlayer thickness was 2 cm, the hydraulic cracks extended to approximately 30% of the cross-section in the coal layer, whereas when the interlayer thickness was 2 cm, the hydraulic cracks only extended in the roof. It can be observed that the interlayer plays a role in blocking the expansion of hydraulic cracks. First, with the increase in interlayer thickness, the in-situ stress is shared by inlayers, which reduces the stress intensity factor of hydraulic cracks, thus inhibiting the expansion of hydraulic cracks. However, the existence of the interlayer consumes pore fluid, resulting in a decrease in fluid flow and pressure entering the coal layer, making it difficult for hydraulic fractures to enter the coal seam. In addition, the modulus of the interlayer was higher than that of the coal layer, which made crack propagation more difficult. Generally, when the thickness of the interlayer reaches 4m, hydraulic fractures will no longer enter the coal seam easily, so it is suggested that fracturing boreholes should be laid in the interlayer or the coal layer.

**Fig 16:**
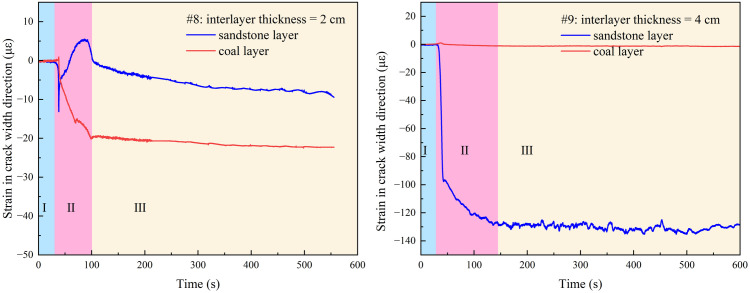
Strain in the direction of the hydraulic crack width at different interlayer thicknesses.

In terms of strain, as shown in Fig 16 (Data is from S5 File), with an increase in the thickness of the interlayer, the tensile strain in the coal layer decreased until there was no obvious tensile deformation, which means that the width of the hydraulic cracks in the coal layer decreased, and the hydraulic fracture did not gradually spread to the coal layer.

**Fig 17:**
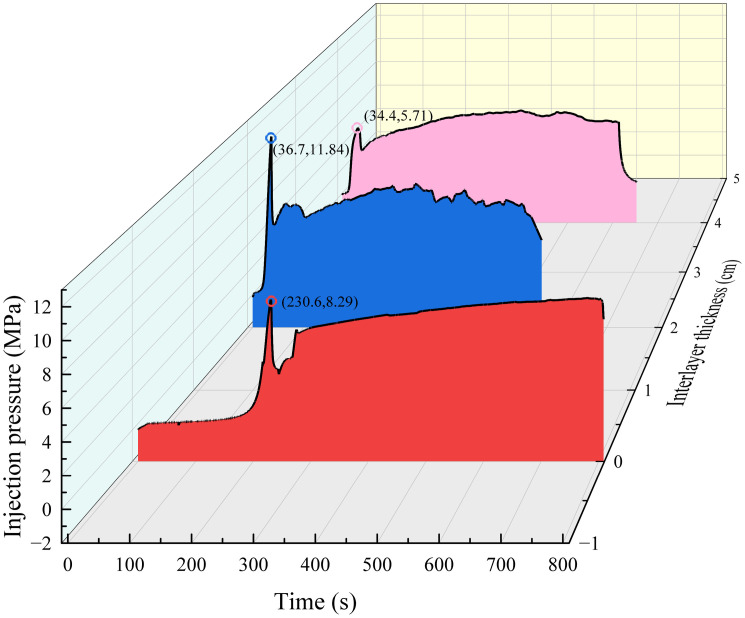
Injection pressures at different interlayer thicknesses.

In the crack initiation stage, it can be observed from sample #8 sample that the existence of an interlayer causes the initiation of hydraulic cracks in the coal seam lag. It is evident that the initiation of cracks in the coal layer leads to the closure of the cracks in the sandstone layer. This is because hydraulic fractures are introduced into the coal layer and quickly share the fracture fluid, which leads to the closure of sandstone layer fractures owing to the decrease in fracture flow in the sandstone layer. This is manifested as a small pressure peak immediately after the pressure peak decreases in the injection pressure, as shown in Fig 17 (Data is from S5 File). With a further increase in the interlayer thickness, the hydraulic fracture can no longer enter the coal seam, and the fracturing fluid is dissipated in the roof only. At the same time, there was no obvious peak value of tensile strain in the roof, which was consistent with the strain of the coal seam in sample #2. This shows that the existence of the interlayer reduces the expansion area of the hydraulic fractures, and the fracture fluid is still sufficient to support smaller hydraulic fractures.

In the fracture closure stage, the existence of an intermediate layer makes the hydraulic fracture closure behavior unclear. In both strain and injection pressure, hydraulic fractures show that cracks are not closed after initiation; in contrast, small cracks are constantly generated and reopened. The existence of the interlayer bears the in-situ stress and dissipates the fracturing fluid, which reduces the fracture propagation area, which is sufficient to continue to support hydraulic fractures and promote the production and reopening of micro-fractures at an injection rate of 60 mL/min.

## 4. Conclusion

In this study, the hydraulic fracture images, deformation of the coal and roof at the interface, and injection pressure curve were analyzed through true triaxial hydraulic fracturing experiments. The effects of flow rate, in-situ stress, coal seam modulus, and interlayer thickness on the fracture propagation width were explored, and the degree of influence of each parameter on the fracture width was analyzed using the normalization method. The main conclusions are as follows.

(1) Hydraulic fracture propagation at the coal-rock interface is complex. Due to the existence of the interface, analysis must integrate multiple variables such as fracture images, strain, and injection pressure.
(2) Greater minimum horizontal stress, coal seam modulus, and interlayer thickness are not conducive to the opening of hydraulic fractures in the coal seam, whereas a greater flow rate is beneficial to the opening of hydraulic fractures in the coal seam. Field operations should optimize fracturing parameters based on actual formation conditions.
(3) The activated area at the interface is unfavorable to the opening of hydraulic fractures in the coal seam, whereas the larger minimum horizontal stress, thickness of the intermediate layer, and modulus of the coal seam are beneficial to the activation of the interface. Therefore, the field design of roof hydraulic fracturing should attempt to reduce the thickness of the interlayer or increase the flow rate to obtain better coal seam fractures.
(4) In the fracture closure stage, the injection pressure fluctuates under the action of the interface and through the fracture, which makes it difficult to distinguish between the reopening of cracks and the generation of new cracks; therefore, it is necessary to combine the strain jump to determine the generation of new fractures.
(5) Hydraulic fractures in coal seams are wider than those in the roof, their widths converge as coal and roof moduli approach each other. Thus, roof fracture width should be considered when selecting proppant sizes.

## Supporting information

S1 File The data of Figs 3 and 4. (RAR)

S2 File The data of Figs 6, 7 and 8. (RAR)

S3 File The data of Figs 9, 10 and 11. (RAR)

S4 File The data of Figs 12, 13 and 14. (RAR)

S5 File The data of Figs 15, 16 and 17. (RAR)

## References

[1] Liang S, Liang Y, Elsworth D, Yao Q, Fu X, Kang J, et al. Permeability evolution and production characteristics of inclined coalbed methane reservoirs on the southern margin of the Junggar Basin, Xinjiang, China. International Journal of Rock Mechanics and Mining Sciences. 2023;171:105581. doi: 10.1016/j.ijrmms.2023.105581

[2] Li Y, Wang Y, Chen B. Oxygen/nitrogen co-doped flexible ultramicroporous carbon monolith with a high CH4 adsorption capacity for CH4/N2 separation from low-concentration coalbed methane. Separation and Purification Technology. 2025;359(P2):130582–130582.

[3] Yan J, Lu Y, Zhong D. Enhanced methane recovery from low-concentration coalbed methane by gas hydrate formation in graphite nanofluids. Energy. 2019;180:728–36. doi: 10.1016/j.energy.2019.180728

[4] Wang K, Wang Y, Xu C. Transition of dominated factors in coal seam gas migration: thermo-hydro-mechanical modelling and analysis. International Journal of Heat and Mass Transfer. 2025;236(P1):126239–126239.

[5] Lv F, Yang R, Gao W, Zhao L, Liu Y, Yan Z, et al. Critical depth prediction based on in-situ stress and gas content model of deep coalbed methane in Liupanshui Coalfield in China. Sci Rep. 2025;15(1):297. doi: 10.1038/s41598-024-84143-3 39747307 PMC11695855

[6] Wang K, Gong H, Wang G. N2 injection to enhance gas drainage in low-permeability coal seam: a field test and the application of deep learning algorithms. Energy. 2024;290:130010.

[7] Ma X, Zhou A, Cheng X. Multi-field coupling models of coal and gas and their engineering applications to CBM in deep seams: A review. Energies. 2024;17(24):6221–6221.

[8] Yu B, Zhang D, Zhao K, Xu B, Geng J, Wang C, et al. Experimental study on stress and permeability response with gas depletion in coal seams. Journal of Natural Gas Science and Engineering. 2022;108:104824. doi: 10.1016/j.jngse.2022.104824

[9] Balucan DR, Turner GL, Steel MK. Acid-induced mineral alteration and its influence on the permeability and compressibility of coal. Journal of Natural Gas Science and Engineering. 2016;973–87.

[10] Esen O, Fişne A. A Comprehensive Study on Methane Adsorption Capacities and Pore Characteristics of Coal Seams: Implications for Efficient Coalbed Methane Development in the Soma Basin, Türkiye. Rock Mech Rock Eng. 2024;57(8):6355–75. doi: 10.1007/s00603-024-03854-1

[11] Moore TA. Coalbed methane: A review. International Journal of Coal Geology. 2012;101:36–81. doi: 10.1016/j.coal.2012.05.011

[12] Yu H, Li Z, Bai Y. Influence of injection pressure on gas adsorption and desorption of anthracite. Energy. 2024;288:129828.

[13] Kang H, Lv H, Gao F, Meng X, Feng Y. Understanding mechanisms of destressing mining-induced stresses using hydraulic fracturing. International Journal of Coal Geology. 2018;196:19–28. doi: 10.1016/j.coal.2018.06.023

[14] Hu Q, Jiang Z, Li Q. Induced stress evolution of hydraulic fracturing in an inclined soft coal seam gas reservoir near a fault. Journal of Natural Gas Science and Engineering. 2021;88(4):103794.

[15] Ni G, Xie H, Li Z. Improving the permeability of coal seam with pulsating hydraulic fracturing technique: a case study in Changping coal mine, China. Process Safety & Environmental Protection. 2018;117:565–72.

[16] Zhao H, Li P, Li X. Fracture propagation and evolution law of indirect fracturing in the roof of broken soft coal seams. International Journal of Coal Science & Technology. 2024;11(1):4–4.

[17] Jing G, Wang Y. Research on Hydraulic Fracturing Pressure Relief and Improvement Permeability Technology of the Soft Coal Seam Roof. ACS Omega. 2024;9(2):2970–9. doi: 10.1021/acsomega.3c09020 38250360 PMC10795046

[18] Li L, Wu W. Variation law of roof stress and permeability enhancement effect of repeated hydraulic fracturing in low‐permeability coal seam. Energy Science & Engineering. 2021;9(9):1501–16.

[19] Li X, Hofmann H, Yoshioka K. Phase-field modelling of interactions between hydraulic fractures and natural fractures. Rock Mechanics and Rock Engineering. 2022;55.

[20] Li Q, Xing H. Influences of the intersection angle between interlayer and in situ stresses during hydraulic fracturing process. Journal of Natural Gas Science and Engineering. 2016;36:963–85. doi: 10.1016/j.jngse.2016.11.032

[21] Kim J, Moridis JG. Numerical analysis of fracture propagation during hydraulic fracturing operations in shale gas systems. International Journal of Rock Mechanics and Mining Sciences. 2015;127–37.

[22] Kar S, Chaudhuri A. Influence of flow and geomechanics boundary conditions on hydraulic fracturing pattern and evolution of permeability between the wells. Engineering Fracture Mechanics. 2024;298109949.

[23] Haifeng Z, Changsong L, Yuangui X. Experimental research on hydraulic fracture propagation in group of thin coal seams. Journal of Natural Gas Science and Engineering. 2022;103.

[24] Wang B, Hou E, Ma L. Research on the Law of Layered Fracturing in the Composite Roof Strata of Coal Seams via Hydraulic Fracturing. Energies. 2024;17(8).

[25] Zhao H, Li P, Li X, Yao W. Fracture propagation and evolution law of indirect fracturing in the roof of broken soft coal seams. Int J Coal Sci Technol. 2024;11(1). doi: 10.1007/s40789-023-00648-8

[26] Liu J, Yang Z, Yi L, Yi D, Li X. Cohesive phase-field model for dynamic fractures in coal seams. International Journal of Mechanical Sciences. 2024;282:109617. doi: 10.1016/j.ijmecsci.2024.109617

[27] Huang B, Zhao X, Shao L. Progressive initiation phenomenon of radial and axial cracks of the borehole during rock hydraulic fracturing. Rock Mechanics & Rock Engineering. 2023;56(11).

[28] Cheng Y, Wu B, Yuan Z. Establishment and application of “T” shape fracture propagation model in hydraulic fracturing of methane well. Journal of China Coal Society. 2013.

[29] Guo T, Tang S, Liu S. Physical simulation of hydraulic fracturing of large-sized tight sandstone outcrops. SPE Journal. 2020;26(1).

[30] Luo J, Shen Y, Meng X, Yang T. Research on the quantitative relationship between stress shadow effect of multiple thick and hard key layers and surface subsidence. Sci Rep. 2025;15(1):508. doi: 10.1038/s41598-024-84179-5 39747521 PMC11695712

[31] Shahoveisi S, Vahab M, Shahbodagh B, Eisenträger S, Khalili N. Phase-field modelling of dynamic hydraulic fracturing in porous media using a strain-based crack width formulation. Computer Methods in Applied Mechanics and Engineering. 2024;429:117113. doi: 10.1016/j.cma.2024.117113

[32] Zhang X, Zhang S, Zou Y, Li J. Effects of laminar structure on fracture propagation and proppant transportation in continental shale oil reservoirs with multiple lithological-combination. Int J Fract. 2025;249(1). doi: 10.1007/s10704-024-00831-1

[33] Yang B, Ma W-J, Pan G-C, Wu K-L, Zhong Y, Chen Z-X. Three-dimensional fracture space characterization and conductivity evolution analysis of induced un-propped fractures in shale gas reservoirs. Petroleum Science. 2024;21(6):4248–61. doi: 10.1016/j.petsci.2024.07.022

[34] Fan J, Liu P, Li J, Jiang D. A coupled methane/air flow model for coal gas drainage: Model development and finite-difference solution. Process Safety and Environmental Protection. 2020;141:288–304. doi: 10.1016/j.psep.2020.05.015

[35] Nordgren RP. Propagation of a vertical hydraulic fracture. Society of Petroleum Engineers Journal. 1970;12(04):306–14.

[36] Geertsma J, De Klerk F. A rapid method of predicting width and extent of hydraulically induced fractures. Journal of Petroleum Technology. 1969;21(12):1571–81.

[37] Shel EV, Paderin GV. Analytical solution of the pseudo-3D model for hydraulic fracturing in a storage-dominated regime. International Journal of Rock Mechanics and Mining Sciences. 2019;114:92–100.

[38] Sakhaee-Pour A, Agrawal A. Integrating acoustic emission into percolation theory to predict permeability enhancement. Journal of Petroleum Science and Engineering. 2018;160:152–9. doi: 10.1016/j.petrol.2017.10.003

[39] Zhang Z, Zhang S, Zou Y. Experimental investigation into simultaneous and sequential propagation of multiple closely spaced fractures in a horizontal well. Journal of Petroleum Science and Engineering. 2021;202(1):108531.

[40] Gage RJ, Wang FH, Fratta D. In situ measurements of rock mass deformability using fiber Bragg grating strain gauges. International Journal of Rock Mechanics and Mining Sciences. 2014;350–61.

